# *Parabacteroides* distinguishes bipolar disorder from schizophrenia: toward a microbial biomarker for differential diagnosis

**DOI:** 10.3389/fmicb.2025.1735998

**Published:** 2026-01-15

**Authors:** Wang Majie, Hu Yifang, Huo Yuncui, Chen Yaping, Li Longhui, Jin Qianyan, Xie Weiwei, Cui Wei, Wang Yucheng

**Affiliations:** 1 Department of Psychiatry, Affiliated Kangning Hospital of Ningbo University, Ningbo, Zhejiang, China; 2 Zhejiang Key Laboratory of Drug Addiction and Brain Health, Ningbo, Zhejiang, China; 3 Ningbo Beilun Third People’s Hospital, Ningbo, Zhejiang, China; 4 Translational Medicine Center of Pain, Emotion and Cognition, Health Science Center, Ningbo University, Ningbo, Zhejiang, China

**Keywords:** bipolar disorder, differential diagnosis, gut-brain axis, *Parabacteroides*, schizophrenia

## Abstract

**Introduction:**

Schizophrenia (SCZ) and bipolar disorder (BD) are severe psychiatric disorders with overlapping clinical manifestations and shared biological mechanisms. Growing evidence implicates the gut microbiota in neuroimmune and metabolic regulation, suggesting a potential role in the pathophysiology and heterogeneity of psychiatric disorders.

**Methods:**

In this comparative study, fecal samples were collected from 43 patients with SCZ, 19 patients with BD, and 40 healthy controls (HC). Gut microbial composition was profiled using 16S rRNA gene sequencing. Microbial diversity, taxonomic composition, and predicted functional pathways were analyzed, and associations between key taxa and clinical symptom dimensions were assessed.

**Results:**

Both SCZ and BD patients exhibited significantly reduced microbial diversity and distinct alterations in gut microbial composition compared with HC. SCZ was characterized by a pro-inflammatory microbial profile, whereas BD showed dysbiosis associated with metabolic disturbances, particularly pathways related to lipid metabolism and obesity susceptibility. Functional prediction revealed downregulation of fatty acid biosynthesis and metabolism pathways, along with selective upregulation of antioxidant-related pathways in SCZ. Notably, *Parabacteroides_B_862066* emerged as a major taxonomic feature distinguishing SCZ from BD. Its relative abundance was negatively correlated with hallucinations and attention impairment in SCZ, but positively associated with negative symptom domains in BD. Integration of *Parabacteroides_B_862066* abundance with symptom scores demonstrated strong discriminative performance between SCZ and BD (AUC = 0.87).

**Discussion:**

These findings suggest that Parabacteroides, particularly *Parabacteroides_B_862066*, may serve as a microbial indicator differentiating SCZ from BD. The distinct microbial and functional profiles observed between the two disorders highlight potential microbiota-related mechanisms underlying psychiatric heterogeneity and may provide insights into subtype-specific pathophysiology.

## 1 Introduction

Schizophrenia (SCZ) and bipolar disorder (BD) are severe, chronic psychiatric disorders characterized by high disability, frequent relapse, and long-term disease progression, leading to profound impairments in patients’ quality of life and social functioning (GBD 2019 Mental Disorders Collaborators, 2022; Owen et al., 2016). SCZ is defined by pervasive disturbances in cognition, affect, and behavior, typified by hallucinations, delusions, and disorganized thought processes. It is among the top contributors to global psychiatric morbidity with an estimated prevalence of about 1% (Liu et al., 2021; McCutcheon et al., 2020). However, BD is characterized by recurrent episodes of mania and depression, where manic phases involve impulsivity, aggression, or risky decision-making, while depressive phases are associated with emotional withdrawal, self-devaluation, and suicidal tendencies. The global prevalence of BD is around 3%, and more than half of the patients experienced suicidal ideation, and about 25% had attempted suicide (Merikangas et al., 2011).

Despite categorical distinctions in diagnostic criteria, growing evidence reveals significant overlap in the endophenotypes and pathophysiological substrates of SCZ and BD (Bourque et al., 2024; Cao et al., 2024; Koshiyama et al., 2020; Li et al., 2020; Cross-Disorder Group of the Psychiatric Genomics Consortium, 2013). Both disorders exhibit psychotic symptoms and cognitive impairments, as well as shared structural and functional abnormalities in the prefrontal cortex, hippocampus, and limbic system (Huang et al., 2020; Koshiyama et al., 2020; Phillips and Swartz, 2014). Dysregulation of immune-inflammatory pathways (Goldsmith et al., 2016), heightened oxidative stress (Jorgensen et al., 2022; Lu et al., 2022; Flatow et al., 2013), metabolic alterations (Needham et al., 2025; Yin et al., 2024), and overlapping genetic risk loci such as *CACNA1C* (Green et al., 2010), further support the hypothesis of shared neurobiological mechanisms. Accordingly, similar pharmacological strategies are often employed in both conditions, including atypical antipsychotics (e.g., olanzapine, quetiapine, risperidone, aripiprazole) and mood stabilizers (e.g., lithium, valproate, lamotrigine). However, prevailing diagnostic protocols are chiefly reliant on symptom-based psychometric tools such as the Positive and Negative Syndrome Scale (PANSS) for SCZ, the Young Mania Rating Scale (YMRS) for manic episodes in BD, and the Hamilton Depression Rating Scale (HAM-D) for depressive episodes in BD. While these instruments index clinical symptomatology, they fail to capture the underlying molecular and neurocircuit dysfunctions, thereby constraining advances in differential diagnosis, stratification, and personalized intervention.

Accumulating evidence from clinical and preclinical studies has increasingly recognized the gut microbiota as a key trans-diagnostic biological factor in major psychiatric disorders (Doenyas et al., 2025; Ju et al., 2023; Ortega et al., 2023). Recent studies have identified shared signatures of gut dysbiosis across these disorders, including reduced microbial diversity; depletion of anti-inflammatory, short-chain fatty acid (SCFA)-producing genera such as *Faecalibacterium* and *Coprococcus*; and enrichment of pro-inflammatory taxa such as *Eggerthella*, *Streptococcus*, and *Lactobacillus* (Cheng et al., 2024; Liu et al., 2024; McGuinness et al., 2022). Notably, such alterations have also been identified in individuals at high risk or in the prodromal phase of severe mental illness (Kong et al., 2025). Mechanistically, the gut microbiota can influence central nervous system function through several pathways, including the production of neuroactive metabolites such as SCFAs and tryptophan-kynurenine derivatives, modulation of peripheral and central immune-inflammatory responses (O’Riordan et al., 2025), microglial activation (Cao et al., 2025), and bidirectional communication with the hypothalamic–pituitary–adrenal (HPA) axis (Loh et al., 2024). These pathways collectively shape neuroinflammation, synaptic plasticity, and monoaminergic signaling, providing biological plausibility for the microbiome’s involvement in mood, cognitive, and psychotic symptoms. Perturbations including diminished microbial diversity, depletion of butyrate-producing taxa, and enrichment of pro-inflammatory microorganisms have also been observed in SCZ and BD cohorts, with additional links reported between antipsychotic responses and microbiome alterations (Vasileva et al., 2024; Zhu et al., 2020; Hu et al., 2016). Nevertheless, the relationships between microbial characteristics and clinical phenotypes, as well as direct comparative studies between SCZ and BD conducted under harmonized recruitment, environmental conditions, and analytical parameters, remain markedly lacking.

To address this gap, the present case–control investigation utilized 16S rRNA gene sequencing and integrated bioinformatic analyses on fecal samples derived from first-episode or recurrent (drug-free ≥ 3 months) patients with SCZ and BD, alongside healthy controls (HC). The primary aim of this study was to identify gut microbial taxa and potential functional pathways specifically associated with SCZ and BD, and to examine their associations with clinical phenotypes, thereby providing insights into disorder-specific microbial signatures and informing the development of microbiome-based auxiliary diagnostic approaches in psychiatric disorders.

## 2 Methods

### 2.1 Participant selection

The study protocols received ethical clearance from the Affiliated Kangning Hospital of Ningbo University (approval number NBKNYY-2022-LC-31) and were registered with the Chinese Clinical Trial Registry (registration number ChiCTR2500114225). The study enrolled 43 patients with SCZ, 19 patients with BD, and 40 healthy controls. All patients were independently diagnosed by two experienced psychiatrists in accordance with the Structured Clinical Interview for the Diagnostic and Statistical Manual of Mental Disorders (DSM-V). The inclusion criteria were: (1) Age between 16 and 65; (2) Patients in their first episode or relapse after ≥ 3 months drug-free; (3) Written informed consent from the participants or their guardians. The exclusion criteria were: (1) Participants with other psychiatric disorders or severe neurological diseases; (2) Participants with severe somatic diseases, infectious diseases, or immune system diseases; (3) Presence of a history of substance abuse; (4) Participants who had taken antibiotics within the past 3 months, or had other medical conditions that could affect gut microbiota composition.

Comprehensive medical histories, physical examinations, and laboratory assessments were systematically recorded for all participants. The Mini-International Neuropsychiatric Interview (MINI) was administered to screen for coexisting psychiatric conditions. Symptom severity and profiles were evaluated using the Scale for the Assessment of Negative Symptoms (SANS) and the Scale for the Assessment of Positive Symptoms (SAPS). Detailed SANS and SAPS scores are summarized in Supplementary Tables S1, S2. Information on medication status at the time of sampling, medication duration, and duration of illness is also provided in these tables.

### 2.2 Fecal sample collection

Fecal specimens were obtained from patients and healthy controls recruited at the Affiliated Kangning Hospital of Ningbo University. All participants provided written informed consent prior to sample collection. Fecal samples from healthy controls were collected at baseline, whereas samples from patients were collected at the time of the first bowel movement after hospital admission, once their clinical condition had stabilized. Samples were collected using sterile containers pre-filled with DNA stabilization buffer and were transported to the laboratory within 30 min after defecation to ensure standardized sample handling. Upon arrival, all samples were immediately stored at −80 °C until further analysis. Sample collection, handling, and storage procedures were consistent across all participants to minimize technical variability. Sequencing was performed by Wekemo Tech Group Co., Ltd. (Shenzhen, China).

### 2.3 DNA extraction and PCR amplification

Total genomic DNA was isolated from fecal samples utilizing the CTAB extraction protocol. DNA concentration and purity were assessed via 1% agarose gel electrophoresis. Subsequently, genomic DNA was diluted to a final concentration of 1 ng/μL using nuclease-free water. The V4 hypervariable region of the bacterial 16S rRNA gene was amplified employing the primer pair 515F (5′-GTGCCAGCMGCCGCGGTAA-3′) and 806R (5′-GGACTACHVGGGTWTCTAAT-3′). Each PCR reaction consisted of 15 μL Phusion® High-Fidelity PCR Master Mix (New England Biolabs), 2 μM of forward and reverse primers, and approximately 10 ng of template DNA. Thermal cycling conditions included an initial denaturation at 98 °C for 1 min, followed by 30 cycles of denaturation at 98 °C for 10 s, annealing at 50 °C for 30 s, and extension at 72 °C for 30 s, with a final extension at 72 °C for 5 min. Equal volumes of TAE buffer were mixed with PCR amplicons, followed by analysis on 2% agarose gel electrophoresis for size verification. PCR products were pooled in equimolar ratios and purified using the Universal DNA Purification Kit (TianGen, China).

### 2.4 Libraries generated and Illumina sequencing

Sequencing libraries were constructed using the NEB Next® Ultra DNA Library Prep Kit for Illumina (New England Biolabs, USA) in accordance with the manufacturer’s protocol, and index barcodes were incorporated during library preparation. Library quality control was conducted on the Agilent 5,400 (Agilent Technologies Co. Ltd., USA). Sequencing was executed on an Illumina platform generating 250 bp paired-end reads.

### 2.5 Data processing

All bioinformatic analyses were performed in QIIME2 (Bolyen et al., 2019; https://docs.qiime2.org), with supplementary analyses executed via custom pipelines. Raw FASTQ files were imported into QIIME2-compatible formats using the qiime tools import plugin. Quality control was conducted prior to downstream analyses. Quality assessment included sequencing depth, the proportion of high-quality bases (Q20/Q30), and the percentage of non-chimeric sequences after denoising. All samples met the predefined QC thresholds (Q30 > 85%; non-chimeric rate > 70%), and therefore no samples were excluded due to quality issues. After processing, the majority of samples yielded approximately 70,000–100,000 non-chimeric reads, and all samples retained more than 50,000 non-chimeric reads. Within the QIIME2 framework, demultiplexed sequences were processed using the DADA2 plugin (*q2-dada2*), which implements the DADA2 algorithm (Callahan et al., 2016) to perform integrated quality filtering, denoising, paired-end merging, and chimera removal, generating amplicon sequence variants (ASVs). Taxonomic assignment of ASVs used the QIIME2 *feature-classifier* plugin (Bokulich et al., 2018) and a pre-trained SILVA 138.2 reference database (Quast et al., 2013; https://www.arb-silva.de), trimmed to the V4 region bound by the 515F/806R primers. Taxonomic notations (e.g., *Parabacteroides_B_862066*) adhere to SILVA conventions, with alphanumeric identifiers representing unique ASVs. Extraneous mitochondrial and chloroplast reads were excluded using the QIIME2 *feature-table* plugin. Following ASV table construction, low-abundance features were further filtered by retaining only ASVs present in at least 20% of samples and with read counts greater than 10, in order to reduce sequencing noise and the influence of extremely rare features. Prior to downstream analyses, ASV counts were normalized using Total Sum Scaling (TSS), whereby read counts for each ASV were divided by the total number of reads per sample to obtain relative abundances.

### 2.6 Statistical analysis

Categorical variables were analyzed using the chi-squared test. Normality of continuous variables was assessed with the Shapiro–Wilk test. For two-group comparisons, the independent-samples t-test was applied to normally distributed data, whereas the non-parametric Mann–Whitney *U* test was used for non-normally distributed data. For comparisons among three groups, one-way analysis of variance (ANOVA) was used for normally distributed data, and the Kruskal-Wallis *H* test was used otherwise. Microbiome data analysis was conducted in R (version 4.5.1; https://www.r-project.org/) and QIIME2. Rarefaction analysis using QIIME2’s *alpha-rarefaction* plugin was performed to verify sufficient sequencing depth, based on observed ASVs, Shannon diversity index, and Faith’s phylogenetic diversity index. Diversity metrics were calculated with the *core-diversity* plugin in QIIME2. *α*-diversity indices (observed ASVs, Chao1, Shannon, and Faith’s phylogenetic diversity indices) were calculated to characterize within-sample diversity. *β*-diversity metrics, including Bray-Curtis, unweighted UniFrac, and weighted UniFrac, were determined to assess inter-sample community structure. Principal coordinate analysis (PCoA) and partial least squares discriminant analysis (PLS-DA) were performed using the R packages *ape* (v5.8.1) and *mixOmics* (v6.32.0) to explore the structural differences in gut microbial communities among groups. Tukey’s HSD *post hoc* test was conducted to assess the statistical significance of differential ASVs. Random forest analysis was performed using the R package *randomForest* (v4.7.1) to classify groups and identify discriminatory taxa. The model was constructed with 500 trees (ntree = 500), while all other parameters were kept at default settings. Feature importance was quantified using the MeanDecreaseGini metric. Model stability and performance were evaluated using the out-of-bag (OOB) error estimate, which serves as the algorithm’s built-in internal cross-validation mechanism. No explicit train-test split was applied. In the Random Forest framework, each tree is trained on a bootstrap sample of the data, while the remaining OOB samples are used for validation. The OOB error curve stabilized as the number of trees increased, indicating that the model did not exhibit overfitting. Diagnostic performance was assessed using receiver operating characteristic (ROC) curve analysis with the *ROCR* (v1.0.11) and *pROC* (v1.19.0) R packages; area under the ROC curve (AUC) values of 0.7–0.9 indicated good diagnostic power, and AUC > 0.9 indicated excellent performance. Correlations between microbial taxa and SAPS or SANS scores were visualized using *pheatmap* (v1.0.13) in R. Functional potential profiling of microbial communities was inferred using PICRUSt (Langille et al., 2013) to predict KEGG Ortholog (KO) abundance. Default parameters were applied unless otherwise specified.

## 3 Results

### 3.1 Clinical and sequencing characteristics of the recruited participants

**Figure 1:**
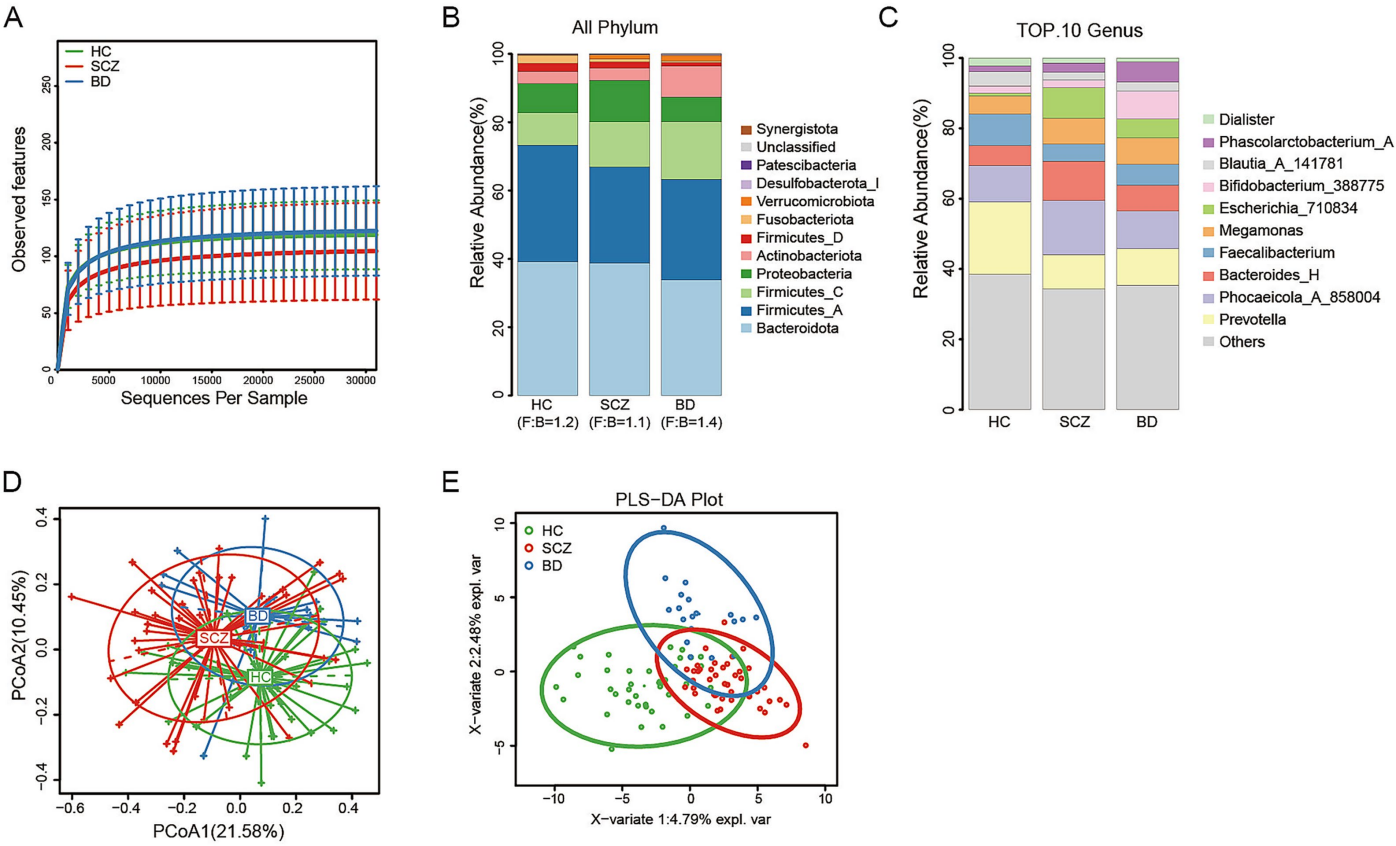
Differential gut microbial characteristics among HC, SCZ and BD groups. **(A)** Rarefaction curves showing sequencing depth and diversity saturation across the three groups. Stacked plots are used to visualize the composition of the phylum **(B)** and genus **(C)** levels. **(D)** Principal coordinate analysis (PCoA) displaying the overall *β*-diversity differences among groups. **(E)** Partial least squares discriminant analysis (PLS-DA) showing the compositional separation trends. These analyses are exploratory and are intended to visualize global community patterns.

According to the inclusion and exclusion criteria, a total of 40 healthy controls, 43 SCZ patients, and 19 BD patients were ultimately included in the study. The HC, SCZ, and BD cohorts were matched for principal demographic variables, including age (H = 1.445, *p* = 0.49), sex (χ^2^ = 0.302, *p* = 0.86), body mass index (BMI, H = 0.146, *p* = 0.93), and fasting blood glucose (FBG, H = 3.824, *p* = 0.15). Comprehensive demographic and clinical details are provided in Table 1. High-quality sequencing reads underwent denoising and ASVs clustering via the DADA2 algorithm in QIIME2. Sequencing depth adequacy was verified by rarefaction curves constructed from ASV counts per sample (Figure 1A), which plateaued, indicating sufficient depth to capture core microbial diversity. Relative abundance analysis at the phylum level (Figure 1B) demonstrated reduced *Firmicutes_A* and *Fusobacteriota* in the patient groups, and elevated levels of *Firmicutes_C*, *Verrucomicrobiota*, and *Desulfobacterota_I*. Notably, *Proteobacteria* was enriched in the SCZ group, while *Actinobacteriota* predominated in BD. *Bacteroidota*-to-*Firmicutes* ratios (F: B) were similar between SCZ (F: B = 1.1) and HC (F: B = 1.2), but elevated in BD (F: B = 1.4). At the genus level (Figure 1C), patients exhibited reduced *Prevotella*, *Faecalibacterium*, *Blautia_A_141781*, and *Dialister*, with increased *Bacteroides_H*, *Megamonas*, *Escherichia_710834*, and *Phascolarctobacterium_A*. *Bifidobacterium_388775* and *Phascolarctobacterium_A* were highly abundant in BD, while *Phocaeicola_A_858004* and *Escherichia_710834* were elevated in SCZ. These genus-level shifts, corroborating phylum-level trends, implicate a pro-inflammatory gut microbiome in SCZ and a distinct metabolic dysbiosis in BD characterized by increased energy metabolism and heightened risk of metabolic syndrome and obesity. Detailed taxonomic profiles are provided in Supplementary Tables S3–S4 and Supplementary Figure S1. PCoA showed stratified separation between HC and patient groups (Figure 1D), while PLS-DA differentiated SCZ from BD, reinforcing divergence in gut community structure among cohorts (Figure 1E).

**Table 1:** Comparison of clinical characteristics among three groups.

Demographic and clinical indexes	HC (*n* = 40)	SCZ (*n* = 43)	BD (*n* = 19)	H/*χ*^2^ value	*p*-value
Age (years; mean± SD)^a^	30.63 ± 8.70	29.14 ± 10.88	29.84 ± 10.96	H = 1.445	0.49
Sex (male/female)^b^	25/15	29/14	13/6	*χ*^2^ = 0.302	0.86
BMI (kg/m2, mean ± SD)^a^	22.72 ± 3.96	23.31 ± 4.74	22.85 ± 3.74	H = 0.146	0.93
FBG (mmol/L, mean± SD)^a^	5.05 ± 0.75	4.76 ± 0.57	4.58 ± 0.55	H = 3.824	0.15

^a^Kruskal-Wallis H test. ^b^Chi-square test.

### 3.2 Diversity and functional annotation of gut microbiota among HC, BD, and SCZ groups

**Figure 2:**
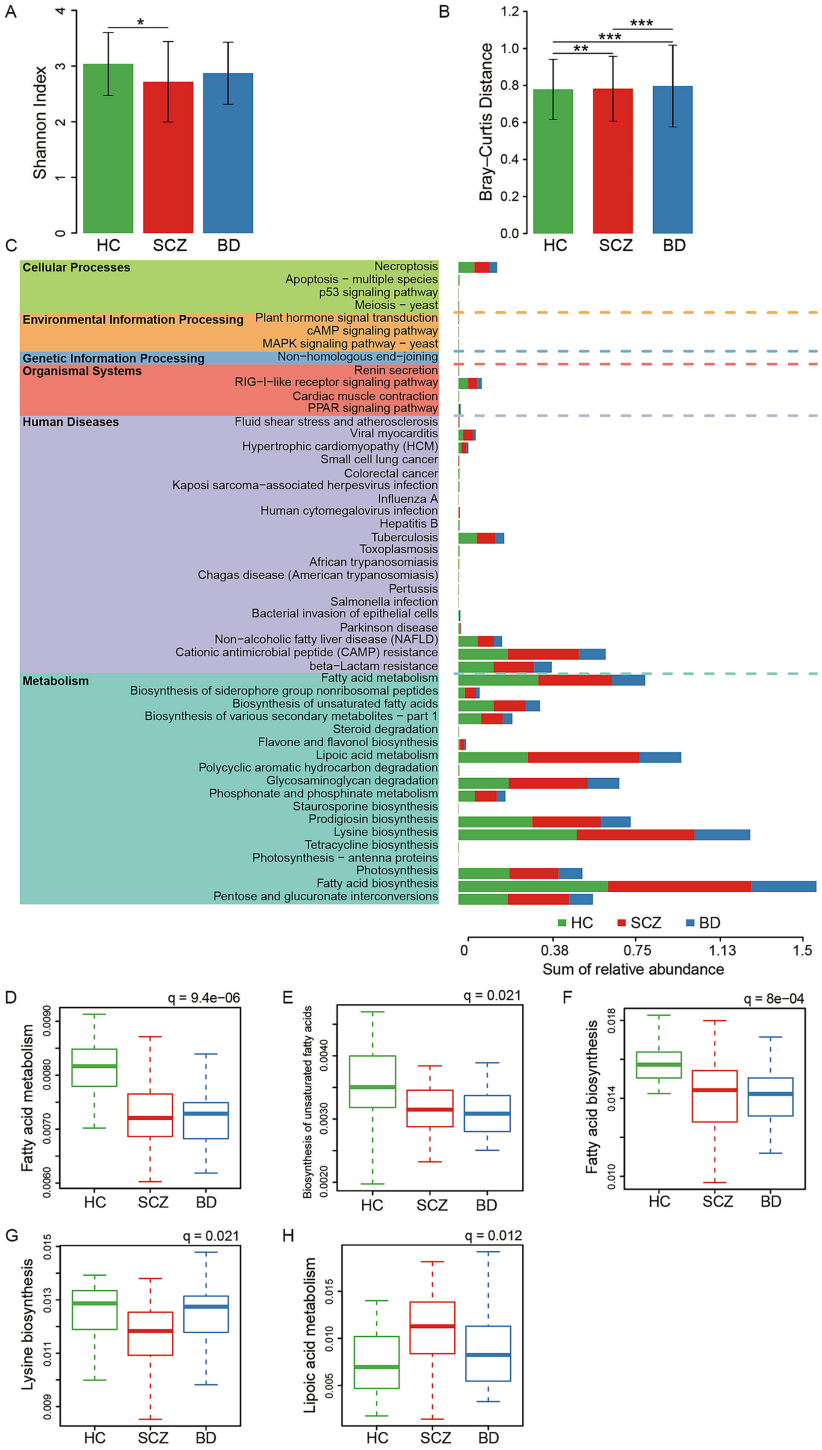
Diversity analysis and functional annotation. **(A)** *α*-Diversity based on the Shannon index. **(B)** β-Diversity measured using the Bray-Curtis distance. **(C)** Functional prediction of bacterial taxa among the three groups based on the KEGG database using PICRUSt2. Statistical significance was assessed using the Kruskal-Wallis *H* test with FDR correction. Box plots of selected pathways are shown: **(D)** Fatty acid metabolism, **(E)** Biosynthesis of unsaturated fatty acids, **(F)** Fatty acid biosynthesis, **(G)** Lysine biosynthesis, and **(H)** Lipoic acid metabolism. For panels **(A)** and **(B)**, statistical significance was determined using the Kruskal-Wallis *H* test. ^*^p < 0.05, **p < 0.01, ***p < 0.001.

**Figure 3:**
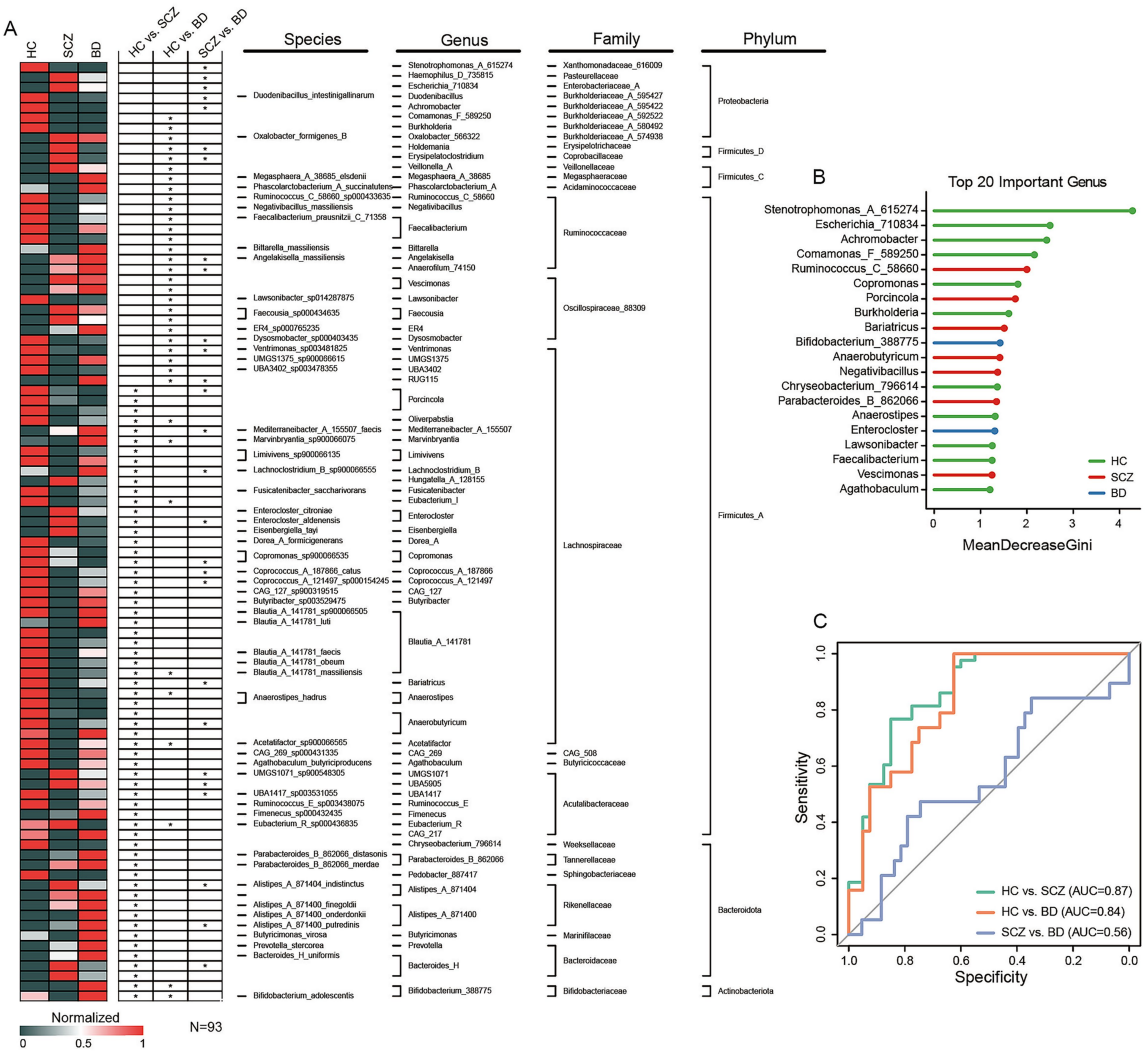
Differential analysis of gut microbiota among HC, SCZ, and BD groups. **(A)** Heatmap displaying 93 ASVs with significant differences, identified using Tukey’s HSD test (*p < 0.05). **(B)** Random forest analysis identifying key genera contributing to group discrimination based on the heatmap results. All genera were ranked by MeanDecreaseGini, and the top 20 taxa with the highest importance values were selected as features. **(C)** ROC curve analysis based on the random forest model.

The *α*-diversity, assessed via the Shannon index (Figure 2A), revealed a downward trend in gut microbial diversity in both SCZ and BD versus HC, with a statistically significant reduction in SCZ (*p* < 0.05), indicative of decreased microbial richness and evenness in psychiatric cohorts and potential ecological destabilization. The *β*-diversity, as evaluated through Bray–Curtis dissimilarity (Figure 2B), highlighted significant differences between patients and HC (*p* < 0.01 for SCZ, *p* < 0.001 for BD), as well as between SCZ and BD (*p* < 0.001), establishing marked dysbiosis in both disorders relative to controls. Functional predictions using PICRUSt2 identified 50 pathways with significant inter-group differences following FDR adjustment (*p* < 0.05), predominantly within Metabolism and Human Diseases categories (Figure 3C). Key metabolic pathways enriched included fatty acid metabolism and biosynthesis (including unsaturated fatty acids), lipoic acid metabolism, and lysine biosynthesis. Both SCZ and BD groups exhibited marked reductions in fatty acid metabolic activities compared to HC, with BD showing the lowest functional output (HC > SCZ > BD; Figures 2D–F), suggestive of attenuated lipid synthesis/oxidation and disrupted cellular energy processes. Conversely, lipoic acid metabolism was significantly upregulated in SCZ (Figure 2H), pointing to a compensatory boost in antioxidative/mithochondrial cofactor pathways. Lysine biosynthesis was selectively diminished in BD but remained stable in HC and SCZ (Figure 2G), potentially compounding metabolic insufficiency given lysine’s role in acetyl-CoA and energy generation. Collectively, these results signal broad downregulation of lipid and amino acid metabolic routes in BD, while SCZ exhibits targeted metabolic adaptations related to antioxidative defense.

### 3.3 Significant differences in gut microbiota among HC, BD, and SCZ groups

High-resolution heatmap analyses across taxonomic ranks (phylum, family, genus, species) revealed 93 significantly altered ASVs among the study groups (Figure 3A). Genus-level random forest modeling delineated the top 20 discriminatory taxa, with *Ruminococcus_C_58660*, *Bifidobacterium_388775*, *Parabacteroides_B_862066*, and *Faecalibacterium* among the most salient (Figure 3B). The random forest-derived ROC curves yielded strong discriminatory power for HC versus SCZ (AUC = 0.87) and HC versus BD (AUC = 0.84), but considerably weaker differentiation between SCZ and BD (AUC = 0.56). Species-level analyses reinforced these findings, identifying *Parabacteroides_B_862066_distasonis, Parabacteroides_B_862066_merdae, Bifidobacterium_adolescentis, Faecalibacterium_prausnitzii_C_71358, Alistipes_A_871400_onderdonkii, and Alistipes_A_871400_putredinis* as high importance features (Supplementary Figures S2A,B). These results corroborate substantial compositional divergence in gut microbiota between patient and control cohorts, yet underscore the limited taxonomic distinction between SCZ and BD at lower ranks.

### 3.4 Diagnostic performance of *Parabacteroides_B_862066* combined with SANS and SAPS in distinguishing between SCZ and BD

**Figure 4:**
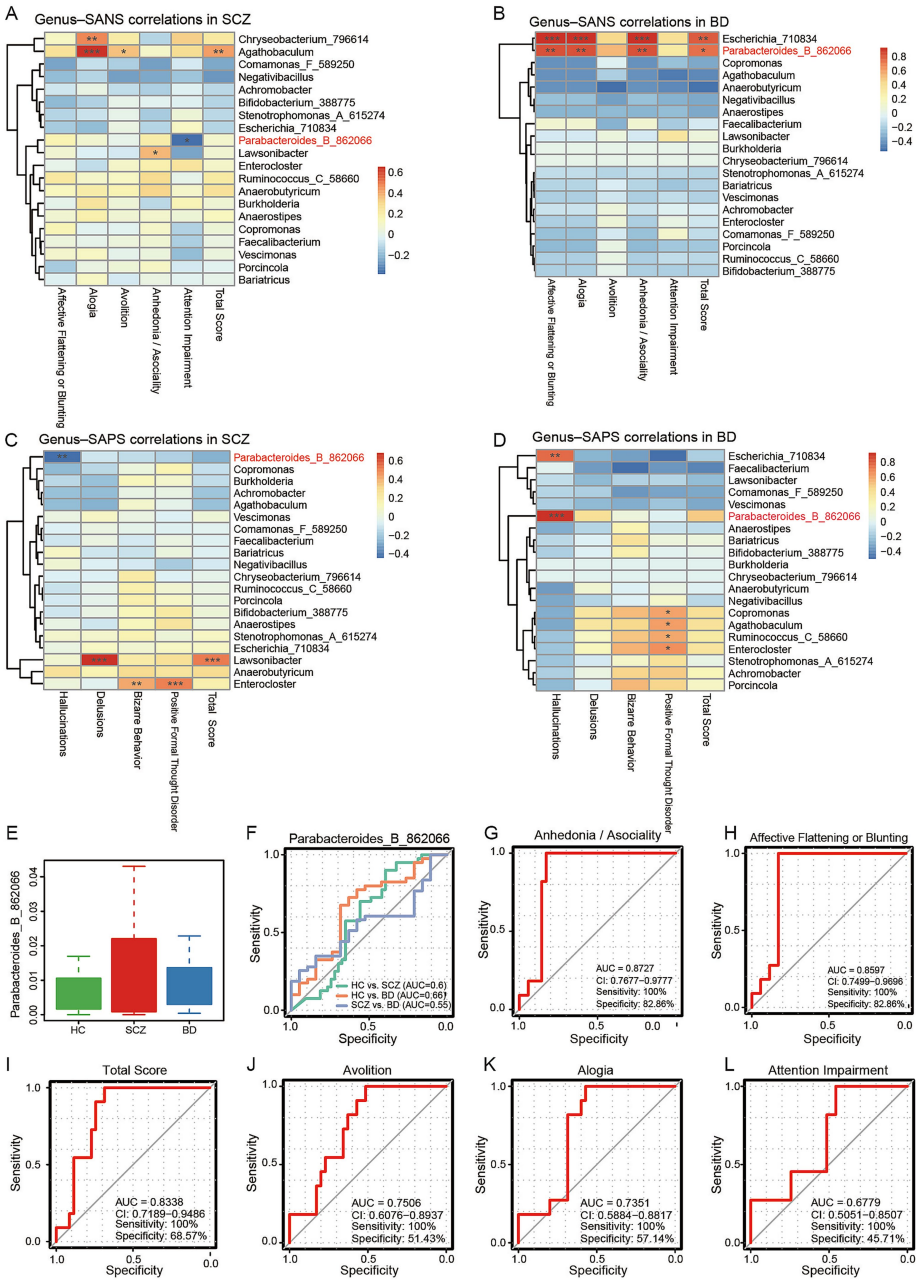
Diagnostic comparison between SCZ and BD based on the combination of positive and negative symptom scales. **(A–D)** Pearson’s correlation heatmaps showing associations between the top 20 genera identified by the random forest model and the SANS and SAPS subscale in SCZ and BD, respectively. **(E)** Boxplot of the genus *Parabacteroides_B_862066*. **(F)** ROC analysis of *Parabacteroides_B_862066* among the three groups. ROC curve analyses were performed by combining *Parabacteroides_B_862066* with the hallucinations score from SAPS and the following SANS subscales: **(G)** Anhedonia/Asociality, **(H)** Affective flattening or blunting, **(I)** Total score, **(J)** Avolition, **(K)** Alogia, and **(L)** Attention impairment. *p < 0.05, **p < 0.01, ***p < 0.001.

Clinical ratings for positive and negative symptoms (SAPS and SANS) were correlated with the top 20 genera from the random forest analysis (Figures 4A–D). *Parabacteroides_B_862066* exhibited diametric correlations in SCZ and BD: in SCZ, its abundance was negatively correlated with hallucination severity (r = −0.45, *p* < 0.01) and attention deficits (r = −0.39, *p* < 0.05), whereas in BD it was strongly positively correlated with hallucinations (r = 0.92, *p* < 0.001) and multiple SANS subdomains—affective flattening/blunting, alogia, anhedonia/asociality (all r = 0.82, *p* < 0.01)—as well as overall SANS score (r = 0.73, *p* < 0.05). Relative abundance patterns are visualized in Figure 4E. ROC analysis indicated moderate diagnostic performance using *Parabacteroides_B_862066* alone: AUC = 0.60 (HC vs. SCZ), 0.66 (HC vs. BD), and 0.55 (SCZ vs. BD) (Figure 4F). Subordinate species results are provided in Supplementary Figures S3A–D. Among these, the SANS anhedonia/asociality subscale demonstrated the best discriminative performance (AUC = 0.8727, sensitivity = 100%, specificity = 82.86%; Figure 4G). In addition, the affective flattening or blunting subscale (AUC = 0.8597; Figure 4H), total SANS score (AUC = 0.8338; Figure 4I), avolition (AUC = 0.7506; Figure 4J), alogia (AUC = 0.7351; Figure 4K), and attention impairment (AUC = 0.6779; Figure 4L) also exhibited varying degrees of discriminative ability.

## 4 Discussion

In this investigation, we conducted a systematic comparison of gut microbiota among HC, SCZ, and BD, ensuring homogeneity in environmental conditions, inclusion criteria, and sequencing protocols. The principal outcomes were as follows: (1) Both SCZ and BD cohorts exhibited diminished microbial diversity and significant alterations in microbial community structure relative to HC; (2) SCZ was characterized by a pro-inflammatory microbiota signature, whereas BD demonstrated a dysbiotic profile aligned with metabolic perturbations, including enhanced energy metabolism and elevated obesity susceptibility; (3) Functional pathway analysis predicted a downregulation of fatty acid metabolic and biosynthetic processes in both illnesses, with a more marked reduction in BD, while SCZ showed selective upregulation of antioxidant-associated metabolic routes; (4) The microbiome-informed machine learning classifier effectively distinguished HC from patient populations but lacked discriminatory power between SCZ and BD groups; and (5) Incorporation of *Parabacteroides_B_862066* as a putative microbial biomarker significantly augmented diagnostic accuracy, particularly when integrated with SAPS hallucination and SANS anhedonia/asociality domain scores.

Prior reports on the association between *Parabacteroides* (including lineages closely related to *Parabacteroides_B_862066*) and major psychiatric disorders have yielded heterogeneous findings. Some studies observed enrichment of *Parabacteroides* in patients, linking it to neuroactive metabolism, particularly *γ*-aminobutyric acid (GABA) signaling and altered intrinsic brain activity, suggesting a possible neuroactive or compensatory function, and other reports described reduced abundance of *Parabacteroides*, associated with increased inflammation and impaired metabolic homeostasis (McGuinness et al., 2022; Strandwitz et al., 2019; Bortolato et al., 2017). Inflammation may involve impairment of intestinal barrier integrity, as reflected by increased circulating markers of intestinal permeability and reduced protective markers in the plasma of patients with schizophrenia, which are further associated with systemic inflammatory indices such as the neutrophil-to-lymphocyte ratio (NLR) (Gokulakrishnan et al., 2022). In other diseases, such as obesity and rheumatoid arthritis, the abundance of *Parabacteroides distasonis* is reduced, while supplementation with this bacterium can alleviate systemic inflammation and improve bile acids (BAs) and glucose metabolism (Sun et al., 2023; Wang et al., 2019). These discrepancies may arise from strain-specific functions, host metabolic or inflammatory backgrounds, and external confounders such as medication, diet, and obesity. Functional analyses show that *Parabacteroides distasonis* strains differ markedly in BAs conversion, GLP-1 induction, and macrophage polarization (Wang et al., 2019; Plovier et al., 2017), which may underlie their bidirectional effects across cohorts.

Based on these findings, this study further explores the physiological relevance and potential mechanisms of *Parabacteroides* in SCZ and BD. This genus may exert multiple biological functions that are highly pertinent to psychiatric disorders, including: (1) regulating bile-acid signaling through receptors such as FXR and TGR5, thereby influencing host energy metabolism and inflammatory responses (Sun et al., 2023); (2) SCFAs and succinate, which modulate metabolic homeostasis (Liu et al., 2025); (3) participating in the regulation of neuroactive compounds such as GABA, thereby affecting neuronal excitability (Strandwitz et al., 2019); and (4) enhancing intestinal barrier integrity and immune homeostasis (Duan et al., 2024; Wu, 2023). These mechanisms are directly linked to core pathological features commonly observed in severe mental disorders, including heightened inflammatory activation, increased oxidative stress, mitochondrial dysfunction, and disruptions of neural circuitry.

The SAPS and SANS subscales reflect dysfunctions in discrete neurocircuitry: hallucinations implicate the auditory-language-prefrontal network, affective flattening and anhedonia/asociality pertain to the prefrontal-striatal-limbic reward circuitry, and attentional impairment involves frontoparietal control systems (Keihani et al., 2025; Stuke et al., 2019; Welberg, 2014). The gut-brain axis may modulate these functional circuits via: (1) Immune-inflammatory signaling, where cytokines such as IL-6 and TNF-*α* alter microglial activation and synaptic plasticity (Fernandes et al., 2016); (2) Microbial metabolites and metabolism-related pathways, including SCFAs, GABA, tryptophan-kynurenine derivatives and neurotransmitter precursor-related pathways (e.g., the tyrosine-dopamine axis), can act on astrocytes and neurons, influencing the blood–brain barrier (Thirion et al., 2023; Strandwitz et al., 2019; Agus et al., 2018); and (3) Endocrine regulation via the HPA axis, modulating excitatory-inhibitory balance in the prefrontal-limbic circuitry (Hu et al., 2016; Sandi and Haller, 2015). In our dataset, *Parabacteroides* showed opposite correlations with SAPS/SANS dimensions between disorders, it suggests that this taxon may act as a host-dependent and context-dependent regulatory factor. In other words, *Parabacteroides* may function as context-dependent modulators within the inflammation-metabolism-neurocircuit axis, providing new insight into the gut-brain mechanisms of severe psychiatric disorders (Sun et al., 2023; McGuinness et al., 2022; Zhu et al., 2020; Wang et al., 2019; Ghaisas et al., 2016). In SCZ, where inflammatory burden and oxidative stress are markedly elevated, certain *Parabacteroides* strains may exert compensatory and protective influences by modulating anti-inflammatory and antioxidant pathways and reducing pro-inflammatory signaling. This could explain their negative associations with positive and cognitive symptom dimensions such as hallucinations and attentional impairment (Sun et al., 2023; Lu et al., 2022; Goldsmith et al., 2016). In contrast, in BD, where metabolic dysregulation is more prominent and reward-related circuits are more severely compromised, *Parabacteroides* may act through GABA metabolism, bile-acid receptor signaling (FXR/TGR5), succinate production, and other metabolic pathways, thereby influencing reward- and motivation-related neural circuits. These mechanisms may underlie its positive associations with the severity of negative symptom dimensions in BD (Strandwitz et al., 2019; Agus et al., 2018). Although incorporating *Parabacteroides* with clinical dimensions improved discriminatory power, its stand-alone AUC of 0.56 for distinguishing SCZ from BD remains limited. Several factors may contribute to this. (1) Substantial biological overlap between SCZ and BD, including immune activation, oxidative stress, and metabolic impairment, is common to both disorders and produces partially shared microbial signatures. (2) Similar lifestyle and environmental influences, such as diet, sleep disruption, and chronic stress, which can shape the microbiome in comparable ways. (3) The limited taxonomic resolution of 16S sequencing, which cannot resolve strain-level heterogeneity, is an important limitation because strain-specific functions, rather than genus- or species-level taxa, are more likely to drive neuroimmune effects. (4) Cross-diagnostic overlap in symptom-related neural circuits, whereby hallucinations, negative symptoms, and affective disturbances share partially convergent neurobiological substrates across SCZ and BD, leading to overlapping microbial correlates. Taken together, these findings indicate that *Parabacteroides* is better suited to serve as one component of a multimodal diagnostic framework that integrates clinical dimensions, metabolomic features, inflammatory markers, and related factors, rather than as an independent biomarker for distinguishing SCZ from BD.

Several limitations should be acknowledged. First, the sample size was relatively small, necessitating validation in larger independent cohorts. Although patients were drug-free for more than 3 months, the limited sample size prevented from distinguishing first-episode from recurrent cases. In addition, the absence of detailed dietary assessments and key treatment-related information constitutes another important limitation. Together, these factors may introduce heterogeneity, as individuals with recurrent illness may harbor greater cumulative inflammatory and metabolic disturbances, while prolonged prior exposure to psychotropic drugs could impart lasting modifications to the gut microbiota. Consistent with this notion, a previous study developed a model based on gut microbiome features that demonstrated high accuracy in distinguishing drug-naïve SCZ patients from those treated with risperidone (Gokulakrishnan et al., 2023). Moreover, differences in illness chronicity and associated neural circuit reorganization may further contribute to divergent microbial profiles. Future work would benefit from more finely grained subtyping strategies or longitudinal designs to delineate temporal microbiome dynamics across illness stages, thereby facilitating the separation of disease-specific signatures from treatment-related and progressive pathological effects. Second, although 16S rRNA sequencing effectively captures overall microbial community structure, its taxonomic resolution is limited and cannot resolve strain-level differences or directly infer key functional pathways (e.g., SCFA, GABA, or BA metabolism). In addition, functional predictions rely on database-based inference and lack experimental validation. Future work should incorporate higher-resolution multi-omics approaches, including metagenomics, transcriptomics, and targeted metabolomics of pathways such as BAs, GABA, and SCFAs, to obtain more precise functional evidence. Finally, by directly comparing gut microbiota differences between patients with SCZ and BD under harmonized recruitment criteria, environmental conditions, and analytical parameters, this study enhances the comparability and robustness of the findings. Integrating microbiome features with clinical phenotypes further suggests that gut microbial characteristics may help delineate patient subtypes beyond traditional diagnostic categories by reflecting underlying inflammatory, metabolic, or neurocircuit vulnerabilities. Such integration may also support the development of multidimensional decision-support models for treatment selection, response prediction, and risk stratification.

## 5 Conclusion

In summary, this study characterized and compared the gut microbial compositions of HC, SCZ, and BD. *Parabacteroides_B_862066* was identified as a potential microbial biomarker associated with disease status. Integrating such microbiome-derived markers with clinical symptom assessments may enhance diagnostic precision, particularly in cases where SCZ and BD present overlapping features. Beyond diagnostic implications, these findings further support the involvement of the microbiota-gut-brain axis in the pathogenesis of BD and SCZ and provide a foundation for future mechanistic studies. The identified microbial alterations may inform investigations into inflammatory signaling, metabolic dysregulation, and neural circuit vulnerability, thereby linking microbial features to physiologically relevant pathways. Finally, future studies using larger, well-characterized prospective cohorts, longitudinal designs, and multimodal integration of microbiome, immune, metabolic, and neurobiological data will be essential to clarify causal relationships and advance clinically meaningful translation.

## Data Availability

The datasets presented in this study can be found in online repositories. The names of the repository/repositories and accession number(s) can be found in the article/Supplementary material. The datasets presented in this study are publicly available in the National Genomics Data Center (NGDC) Genome Sequence Archive (GSA) repository under the accession number PRJCA049433.
