# Supplementary figures and images for: Parabacteroides distinguishes bipolar disorder from schizophrenia: toward a microbial biomarker for differential diagnosis
Source: Front Microbiol. 2026 Jan 15;16:1735998. doi: 10.3389/fmicb.2025.1735998 (PMC12853643; doi:10.3389/fmicb.2025.1735998)

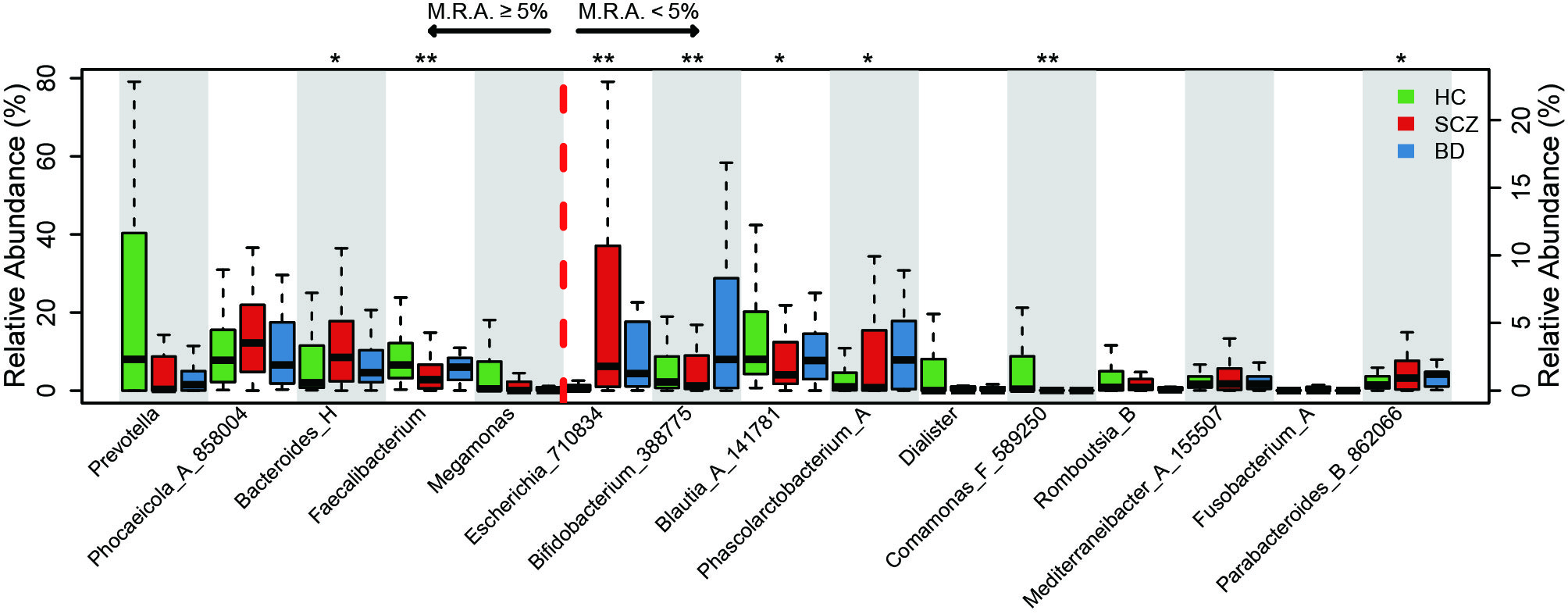

Supplement: Supplementary file 1 [file Image_1.jpeg]

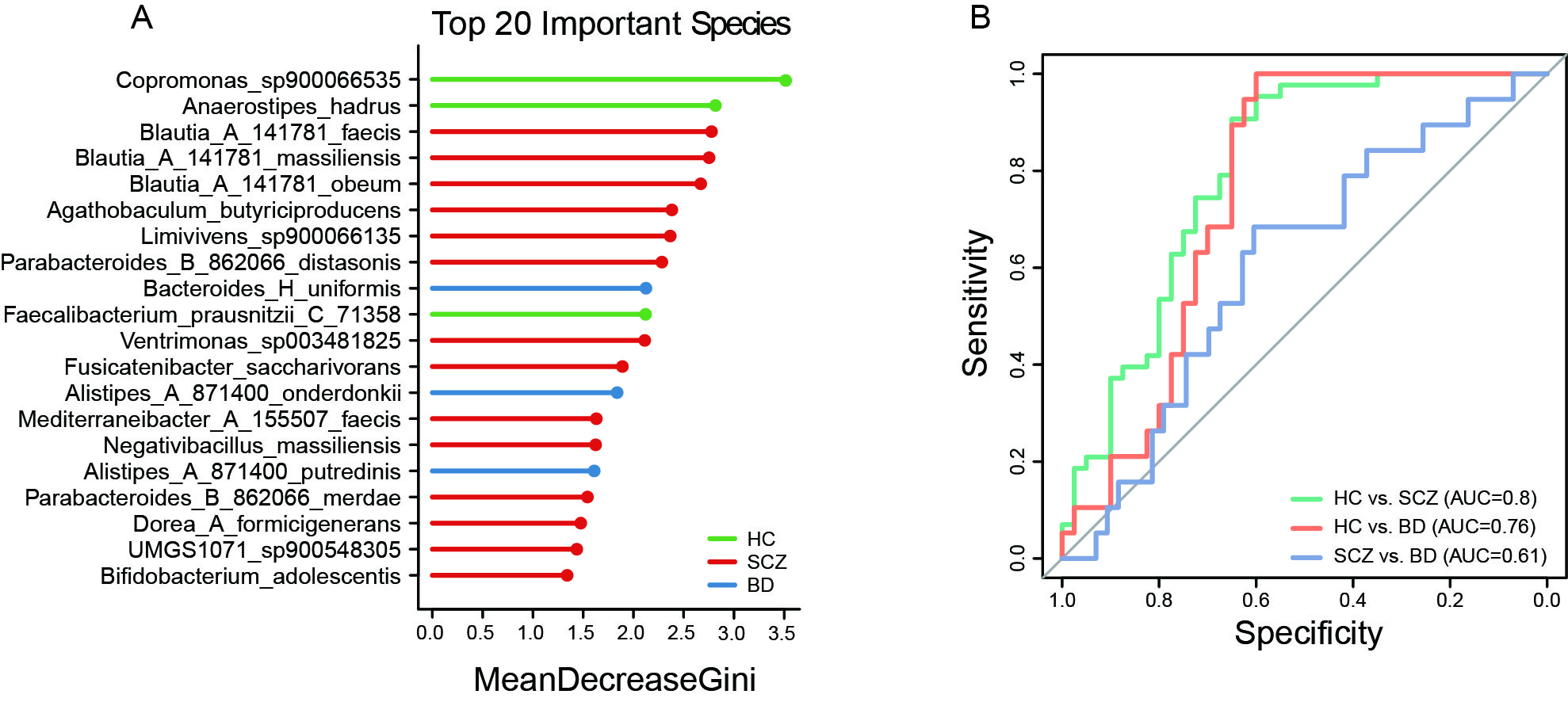

Supplement: Supplementary file 2 [file Image_2.jpeg]

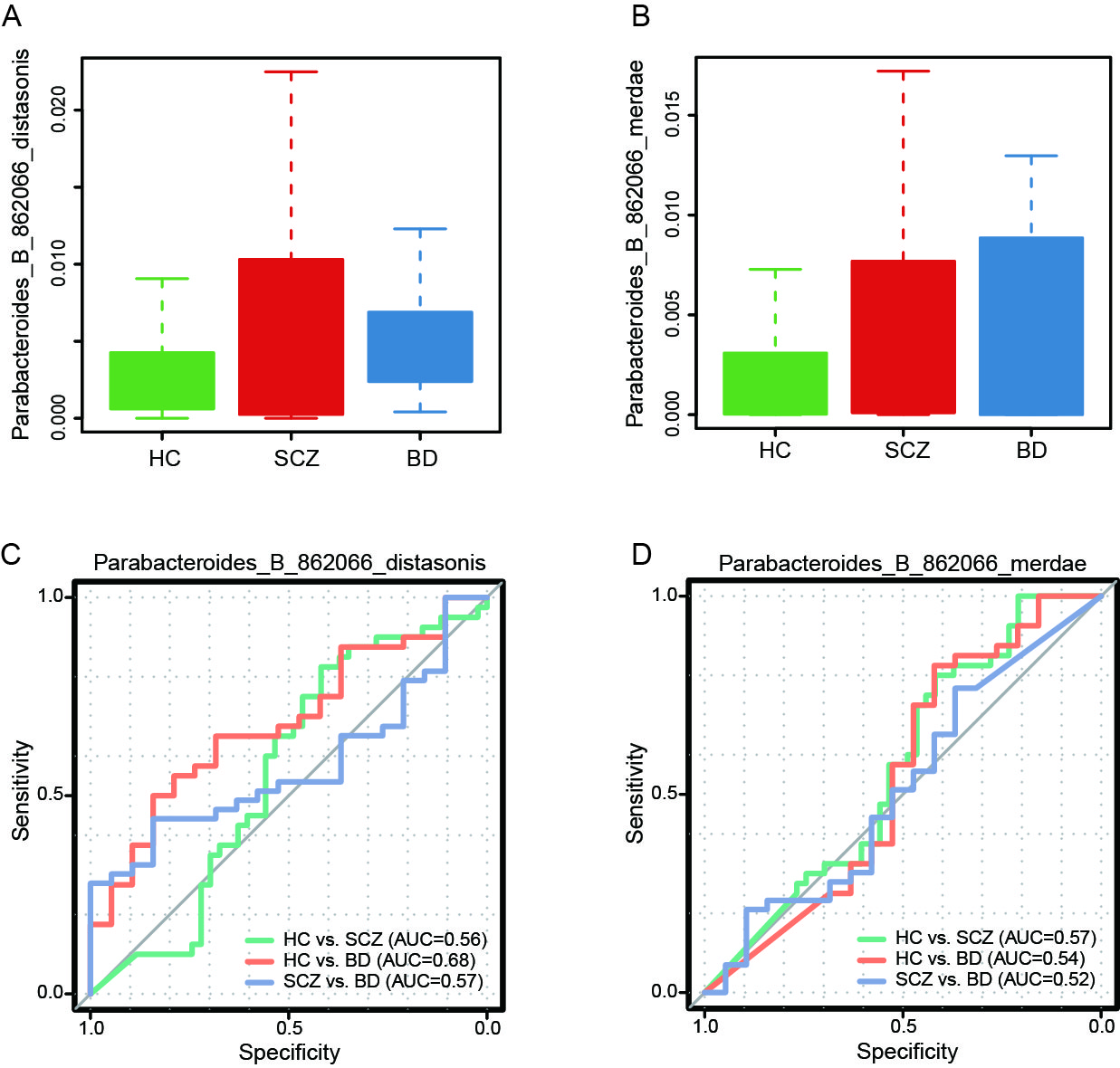

Supplement: Supplementary file 3 [file Image_3.jpeg]
