# Supplementary material for: Parabacteroides distinguishes bipolar disorder from schizophrenia: toward a microbial biomarker for differential diagnosis
Source: Front Microbiol. 2026 Jan 15;16:1735998. doi: 10.3389/fmicb.2025.1735998 (PMC12853643; doi:10.3389/fmicb.2025.1735998)
Supplement: Supplementary file 4 [file Supplementary_file_1.docx]

**Legends for supplementary figures**

**Supplementary Figure S1**. Boxplots showing the relative abundances of the top 15 bacterial genera. Differences among groups were analyzed using ANOVA, **p* < 0.05, ***p* < 0.01, ****p* < 0.001. M.R.A. denotes mean relative abundance.

**Supplementary Figure S2.** Random forest analysis at the species level. (A) Species with significant contributions to group discrimination were identified by random forest analysis based on the heatmap results. (B) ROC curve analysis was performed based on the random forest model.

**Supplementary Figure S3.** Distribution and ROC analysis of *Parabacteroides_B_862066_distasonis* and *Parabacteroides_B_862066_merdae* among three groups. (A) Boxplot of *Parabacteroides_B_862066_distasonis.* (B) Boxplot of *Parabacteroides_B_862066_merdae*. (C) ROC analysis of *Parabacteroides_B_862066_distasonis.* (D) ROC analysis of *Parabacteroides_B_862066_merdae.*
